# Supplementary material for: Revisiting the distribution of oceanic N2 fixation and estimating diazotrophic contribution to marine production
Source: Nat Commun. 2019 Feb 19;10:831. doi: 10.1038/s41467-019-08640-0 (PMC6381160; doi:10.1038/s41467-019-08640-0)
Supplement: Supplementary file 1 — Supplementary Information [file 41467_2019_8640_MOESM1_ESM.pdf]

Tang et al. Revisiting the distribution of oceanic N_2 fixation and estimating diazotrophic contribution to marine production

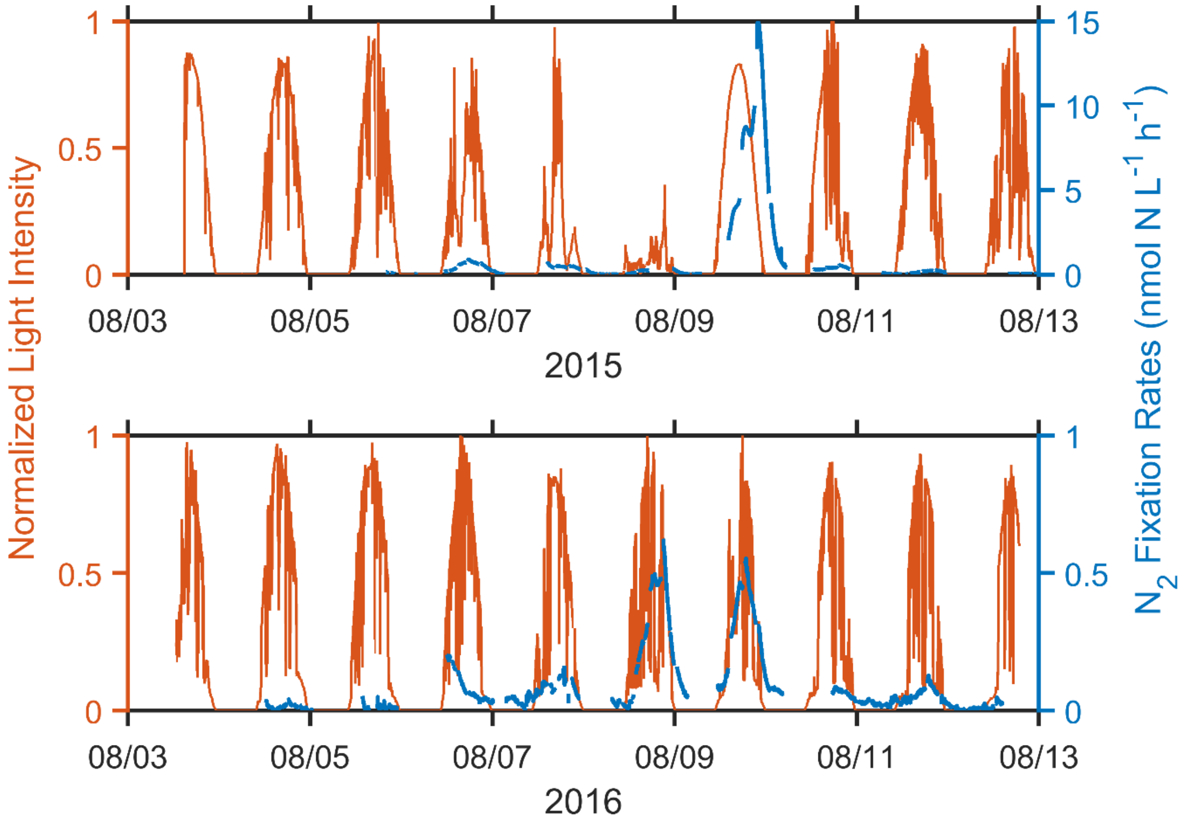

Supplementary Fig. 1| Sunlight intensity (orange) and hourly volumetric N_2 fixation rates (blue) measured during the 2015 (top) and 2016 cruises (bottom). Solar radiation was measured in 2015 while photosynthetically available radiation (PAR) was measured on the 2016 cruise. To account for this difference, light intensity is normalized to the maximum light observed each year as a qualitative proxy for light availability. Note that the range of N_2 fixation rates is different for the 2015 and 2016 cruises.

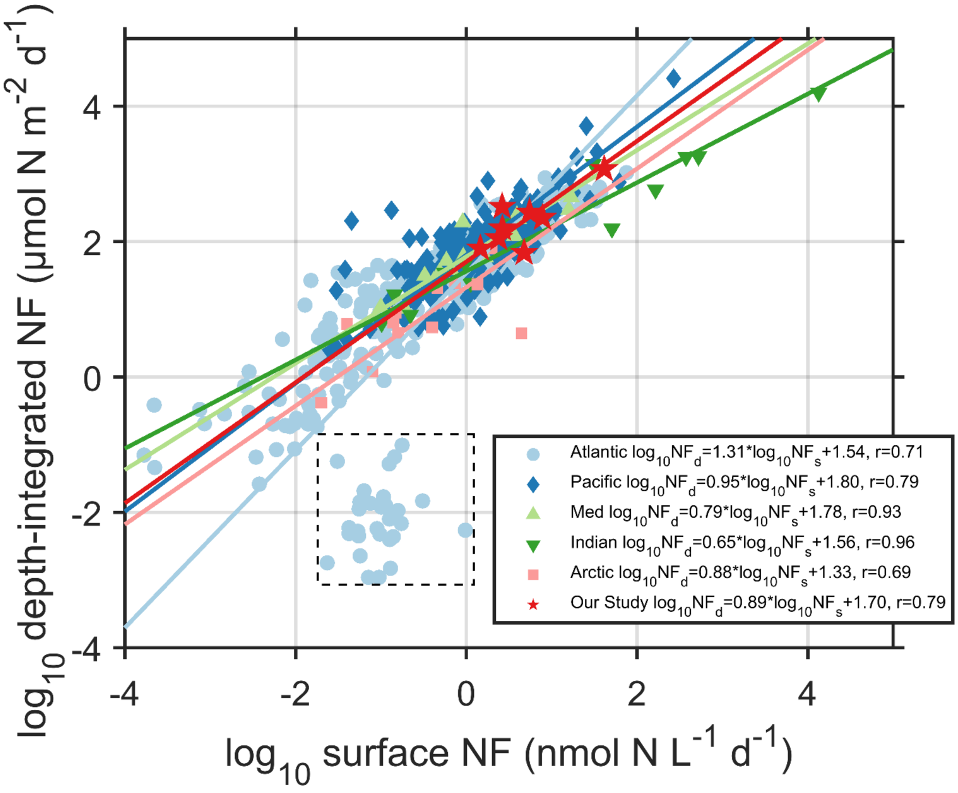

Supplementary Fig. 2| Relationship between depth-integrated N_2 fixation rates (NF_d) and surface volumetric N_2 fixation rates (NF_s). Least Squares Bisector linear regression was applied to observations from various ocean basins and compared to our data collected by discrete $^{15}N_2$ incubation experiments during 2015 cruise. For data from the Atlantic Ocean, the outliers shown in the dashed box may potentially bias the regression analysis. An equation derived from our study is used to convert our underway surface N_2 fixation rates to depth-integrated rates. Data used for this analysis are shown in the Supplementary Data 1.

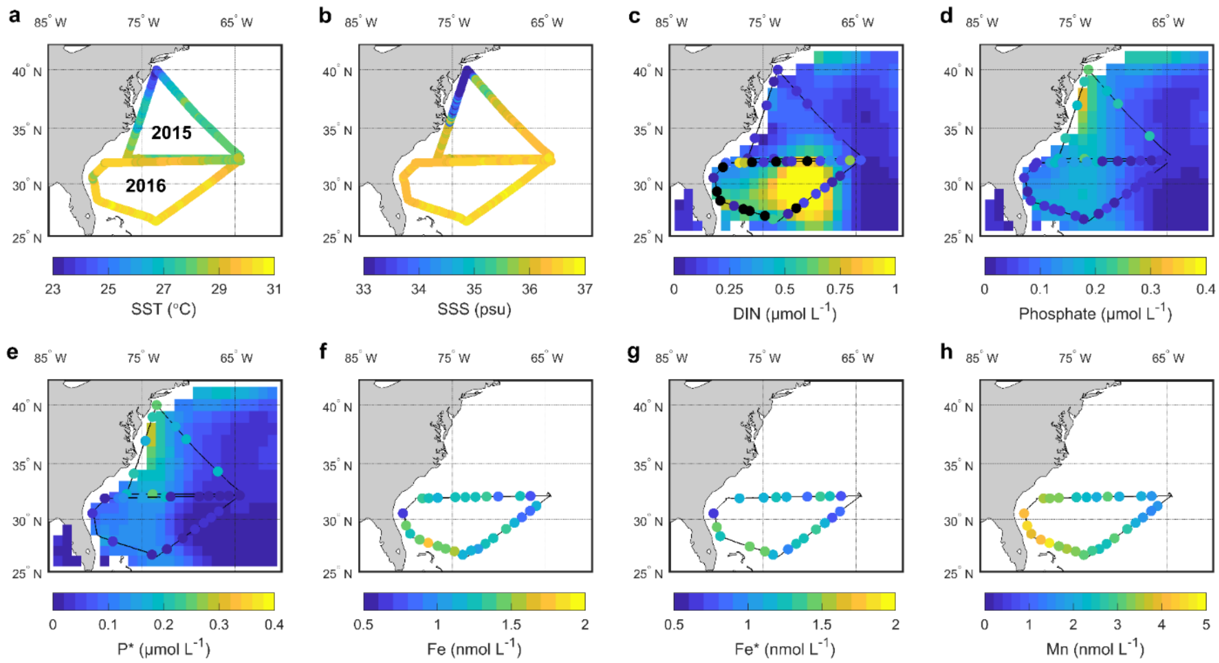

Supplementary Fig. 3| Distribution of various surface (~5 m) properties. Maps of (**a**) sea surface temperature (SST), (**b**) sea surface salinity (SSS), (**c**) dissolved inorganic nitrogen concentrations (DIN = nitrate + nitrite) overlaid on climatology of DIN in August with black markers representing stations below the detection limit, (**d**) phosphate concentrations (P) overlaid on climatology of phosphate in August, (**e**) calculated excess phosphorus (P*) overlaid on climatology of P* in August, (**f**) dissolved iron concentrations (Fe), (**g**) Fe*calculated at stations where both phosphate and Fe concentrations are available, (**h**) dissolved manganese concentrations (Mn). Trace metal data are only available for 2016 cruise.

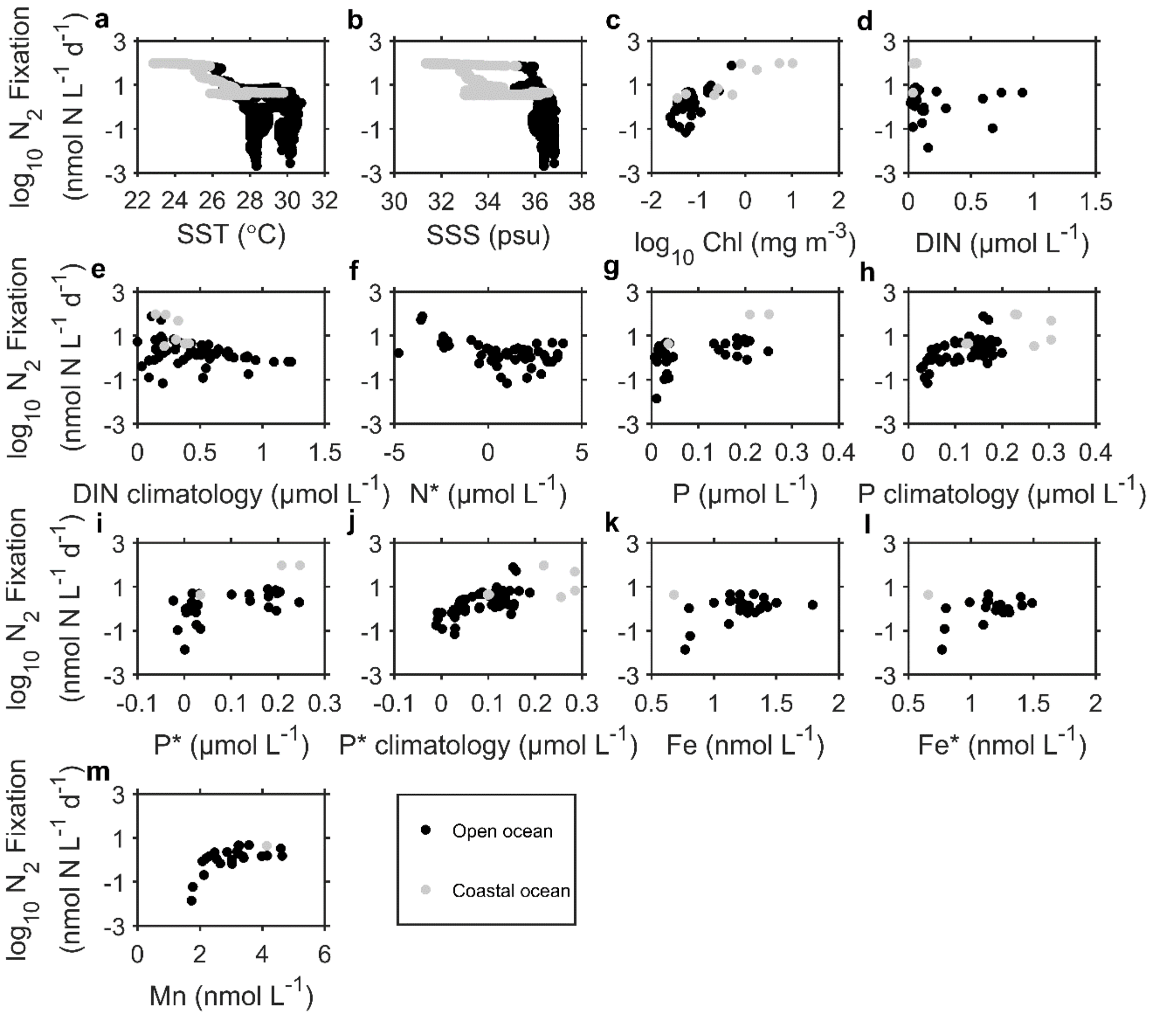

Supplementary Fig. 4| Relationship between surface daily N_2 fixation rates and various properties during the 2015 and 2016 cruises. N_2 fixation rates vs (**a**) SST (r = -0.60†), (**b**) SSS (r = -0.68†), (**c**) satellite observed chlorophyll-*a* concentration (r = 0.80†), (**d**) surface *in-situ* DIN (r = -0.09), (**e**) surface DIN climatology in August (r = -0.38†), (**f**) subsurface N* climatology at δ_θ=26.5 kg m^{-3} in August (r = -0.38†), (**g**) surface *in-situ* P (r = 0.54†), (**h**) surface P climatology in August (r = 0.64†), (**i**) *in-situ* P* (r = 0.60†), (**j**) surface P* climatology in August (r = 0.71†), (**k**) surface *in-situ* Fe (r = 0.33), (**l**) surface *in-situ* Fe* (r = 0.36), (**m**) surface *in-situ* Mn (r = 0.63†). Coastal and open oceans are separated based on -200 m bathymetry. † denotes 95% confidence level in the statistical analyses.

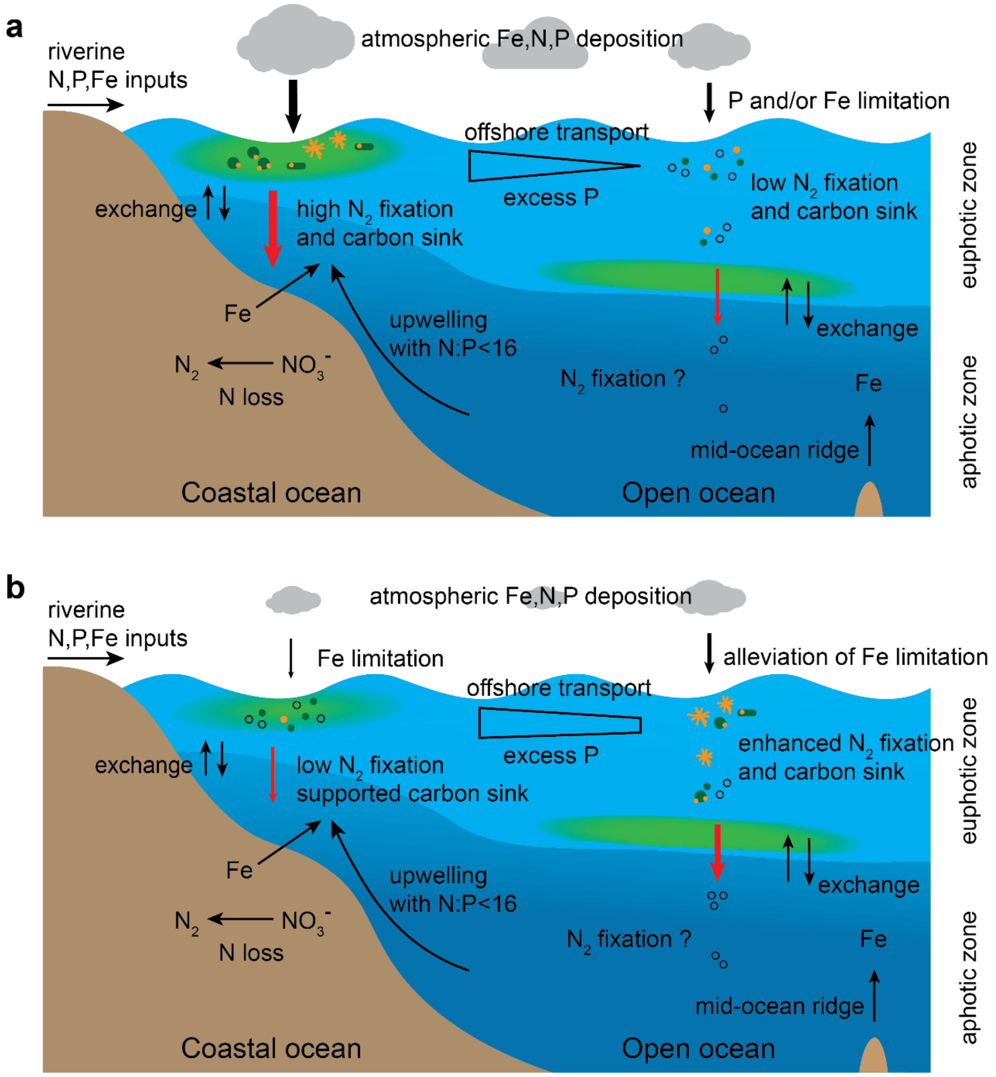

Supplementary Fig. 5| Conceptual diagram of contrasting mechanisms driving N_2 fixation in coastal and open oceans. (**a**) Scenario representative of the western North Atlantic. Excess phosphorus is supplied to the surface in the coastal region from upwelled deep waters with an N:P ratio less than 16 due to denitrification and/or from riverine inputs. The coastal ocean with replete Fe can sustain high N_2 fixation rates under transient nitrogen limitation. According to this scenario, intense N_2 fixation contributes to carbon sequestration (red arrows). Offshore seawater transport with excess phosphorus continues to harbor diazotrophs[1]. Once phosphorus is depleted and/or Fe supply decreases along the trajectory, N_2 fixation is limited. Diazotrophic community composition also shifts along the trajectory. (**b**) Contrasting scenario which may be representative of the Eastern Equatorial Pacific. N_2 fixation in the upwelled waters is hypothesized to be limited due to low Fe concentrations, despite excess phosphorus[2]. When excess phosphorus is transported offshore and if Fe stress is alleviated by episodic atmospheric Fe deposition or by horizontal and vertical Fe inputs, N_2 fixation is stimulated and the carbon sink is enhanced[3]. Note that the low N_2 fixation rates in coastal waters may be also related to other conditions, e.g. lack of conditioned diazotrophs[4], nitrogen nutrients exceeding the threshold inhibiting N_2 fixation. N_2 fixation in the aphotic zone is not discussed in this conceptual diagram.

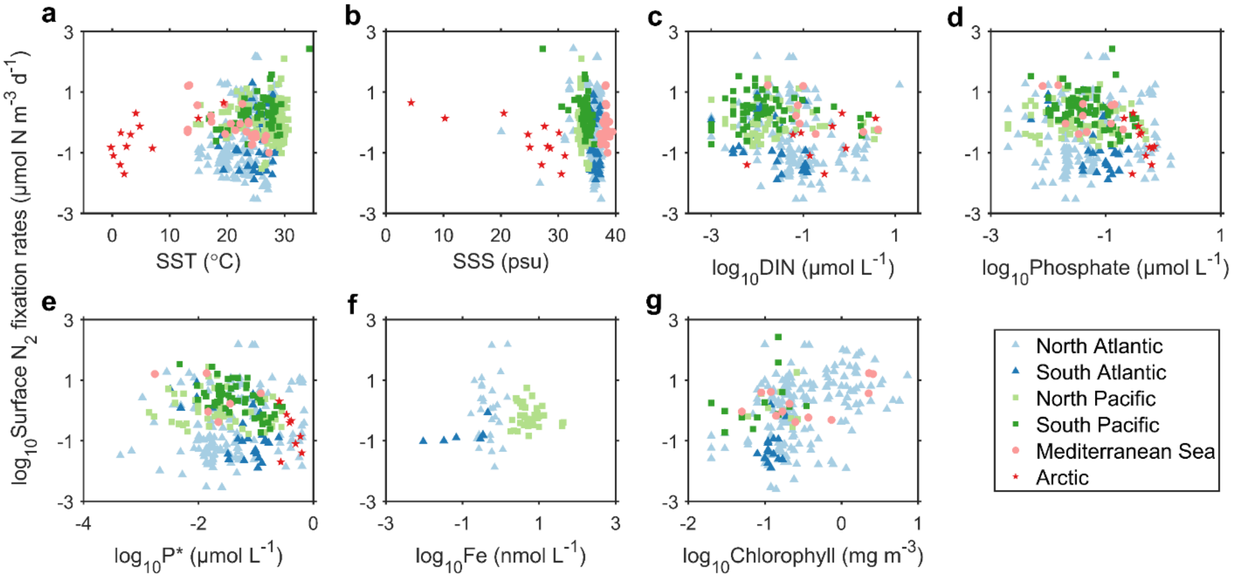

Supplementary Fig. 6| Relationship between surface N_2 fixation rates (≤ 5 m) and various surface properties in the global N_2 fixation dataset. N_2 fixation vs (**a**) SST (r = 0.22†), (**b**) SSS (r = -0.14†), (**c**) DIN (r = -0.30†), (**d**) phosphate (r = -0.08), **(e**) P* (r = -0.05), (**f**) dissolved Fe concentration (r = 0.07), (**g**) chlorophyll-*a* concentration (r = 0.42†). † denotes 95% confidence level in the statistical analyses. Data used for this analysis are shown in Supplementary Data 1.

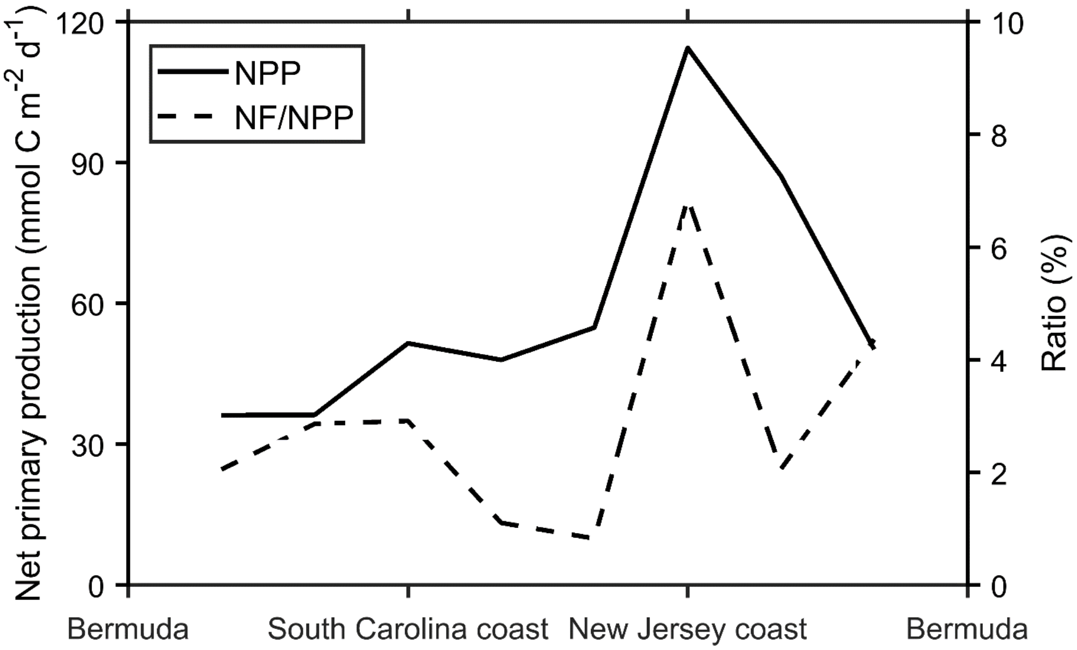

Supplementary Fig. 7| Net primary production and contribution of N_2 fixation to net primary production on the 2015 cruise. Data were collected from discrete dual tracer $^{15}N_2$ and ^{13}C incubations.

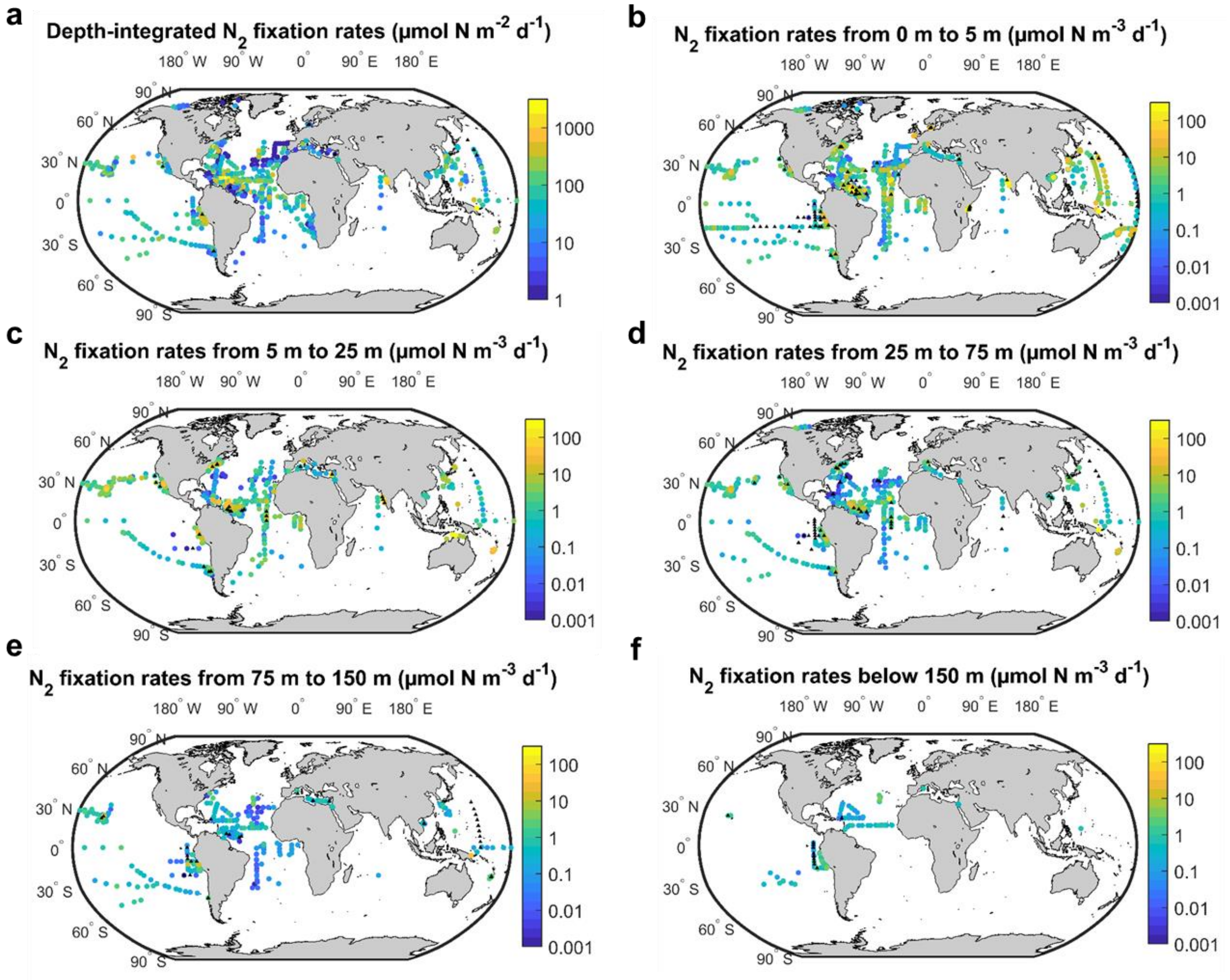

Supplementary Fig. 8| Distribution of N_2 fixation rates in the global ocean. Depth-integrated N_2 fixation rates (**a**) and volumetric N_2 fixation rates from 0-5 m (**b**), 5-25 m (**c**), 25-75 m (**d**), 75-150 m (**e**), and from depths below 150 m (**f**). Zero values are depicted as black triangles. Data used for this analysis are shown in the Supplementary Data 1.

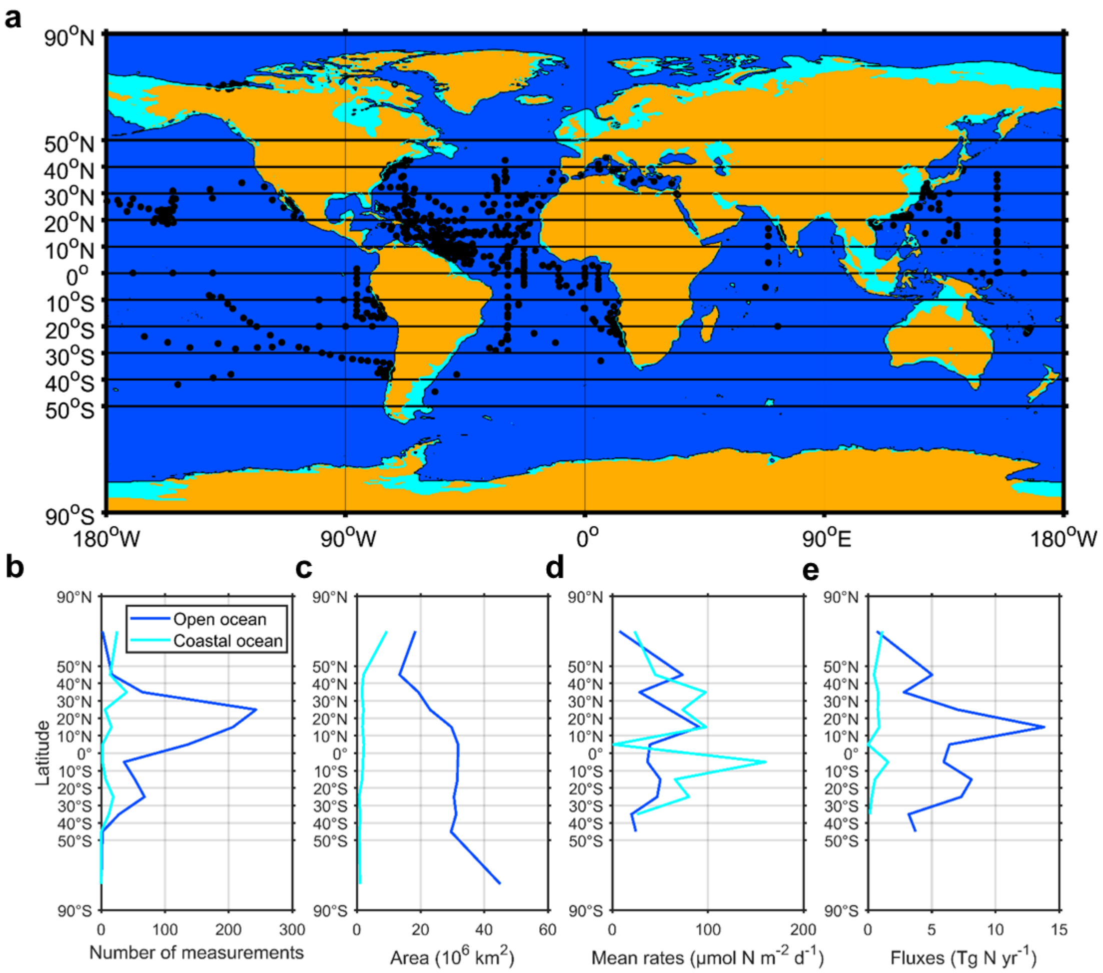

Supplementary Fig. 9| Separation of coastal (cyan) and open oceans (blue) using the -200 m bathymetric contour as a threshold. (**a**) Black horizontal lines show the latitudinal bands used for surface area calculations and the black dots are measurements used to calculate the N_2 fixation budget. Number of measurements (**b**), areal extents (**c**), geometric mean N_2 fixation rates (**d**), and geometric mean fluxes (**e**) of the coastal and open oceans in each latitudinal band.

Supplementary Table 1| N_2 fixation distribution in coastal and open oceans shown in latitudinal bands (standard deviations in parentheses).

Latitudinal bands	Arithmetic				Geometric			
	Rates (μmol N m^{-2} d^{-1})		Fluxes (Tg N yr^{-1})		Rates (μmol N m^{-2} d^{-1})		Fluxes (Tg N yr^{-1})	
	Coastal ocean	Open ocean	Coastal ocean	Open ocean	Coastal ocean	Open ocean	Coastal ocean	Open ocean
>50°N	106.9 (260.9)	7.5 (2.1)	5.2 (12.6)	0.7 (0.2)	23.3 (4.8)	7.4 (1.2)	1.1 (0.2)	0.7 (0.1)
40°N-50°N	90.8 (132.1)	117.5 (122.1)	1.0 (1.4)	8.1 (8.4)	45.0 (3.4)	73.5 (2.7)	0.5 (0.04)	5.0 (0.2)
30°N -40°N	145.2 (116.3)	93.3 (153.3)	1.2 (1.0)	9.2 (15.2)	97.6 (2.8)	28.6 (5.7)	0.8 (0.02)	2.8 (0.6)
20°N -30°N	95.2 (62.4)	151.0 (382.1)	1.0 (0.6)	17.7 (44.9)	73.5 (2.2)	59.9 (4.3)	0.8 (0.02)	7.0 (0.5)
10°N -20°N	397.3 (651.4)	288.9 (570.7)	3.6 (5.9)	43.9 (86.7)	98.1 (5.5)	90.9 (5.6)	0.9 (0.05)	13.8 (0.8)
0°N-10°N	1.2 (0.2)	189.2 (528.1)	0.01 (0)	30.7 (85.8)	1.2 (1.1)	39.4 (6.5)	0.01 (0.01)	6.4 (1.1)
0°S -10°S	184.2 (128.3)	63.4 (75.4)	1.8 (1.3)	10.2 (12.2)	160.2 (1.7)	36.8 (3.1)	1.6 (0.02)	6.0 (0.5)
10°S -20°S	271.9 (299.2)	128.5 (159.2)	2.3 (2.5)	20.8 (25.7)	65.7 (9.5)	50.3 (4.9)	0.6 (0.1)	8.1 (0.8)
20°S -30°S	111.7 (82.7)	156.1 (314.0)	0.4 (0.3)	24.3 (48.9)	80.2 (2.6)	47.0 (5.3)	0.3 (0.01)	7.3 (0.8)
30°S -40°S	50.7 (56.1)	36.5 (46.5)	0.3 (0.3)	5.8 (7.4)	25.8 (3.4)	20.1 (2.8)	0.1 (0.02)	3.2 (0.4)
40°S -50°S	/	54.5 (68.6)	/	8.2 (10.4)	/	24.7 (4.2)	/	3.7 (0.6)
>50°S	/	/	/	/	/	/	/	/

Supplementary References

1 Palter, J. B., Lozier, M. S., Sarmiento, J. L. & Williams, R. G. The supply of excess phosphate across the Gulf Stream and the maintenance of subtropical nitrogen fixation. *Global Biogeochemical Cycles* **25** (2011).

2 Knapp, A. N., Casciotti, K. L., Berelson, W. M., Prokopenko, M. G. & Capone, D. G. Low rates of nitrogen fixation in eastern tropical South Pacific surface waters. *Proceedings of the National Academy of Sciences* **113**, 4398-4403 (2016).

3 Bonnet, S., Caffin, M., Berthelot, H. & Moutin, T. Hot spot of N_2 fixation in the western tropical South Pacific pleads for a spatial decoupling between N_2 fixation and denitrification. *Proceedings of the National Academy of Sciences* **114**, 2800-2801 (2017).

4 Wasmund, N. *et al*. Missing nitrogen fixation in the Benguela region. *Deep Sea Research Part I: Oceanographic Research Papers* **106**, 30-41 (2015).
