# Supplementary material for: Revisiting the distribution of oceanic N2 fixation and estimating diazotrophic contribution to marine production
Source: Nat Commun. 2019 Feb 19;10:831. doi: 10.1038/s41467-019-08640-0 (PMC6381160; doi:10.1038/s41467-019-08640-0)
Supplement: Supplementary file 3 — Description of Additional Supplementary Files [file 41467_2019_8640_MOESM3_ESM.pdf]

Description of Additional Supplementary Files

File Name: Supplementary Data 1
Description: Updated global N_2 fixation database. 1) updated depth-integrated N_2 fixation rates in the global ocean; 2) updated volumetric N_2 fixation rates in the global ocean; 3) references to the data sources.
